# Supplementary material for: Identification of photoperiod-induced specific miRNAs in the adrenal glands of Sunite sheep (Ovis aries)
Source: Front Vet Sci. 2022 Jul 22;9:888207. doi: 10.3389/fvets.2022.888207 (PMC9354845; doi:10.3389/fvets.2022.888207)
Supplement: Supplementary file 1 [file Data_Sheet_1.ZIP › Supplementary file/miRDeep2 file.zip!/miRDeep2 file/Novel_Structure/LP42A1/Novel_172.pdf]

Provisional ID	:	NC_019462.2_12144
Score total	:	1.7
Score for star read(s)	:	-1.3
Score for read counts	:	0
Score for mfe	:	1.4
Score for randfold	:	1.6
Score for cons. seed	:	
Total read count	:	35
Mature read count	:	35
Loop read count	:	0
Star read count	:	0

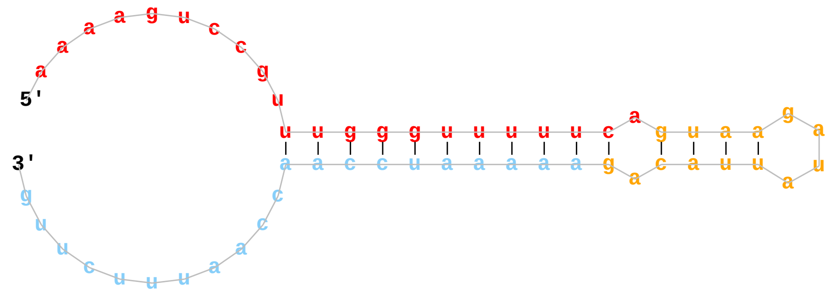

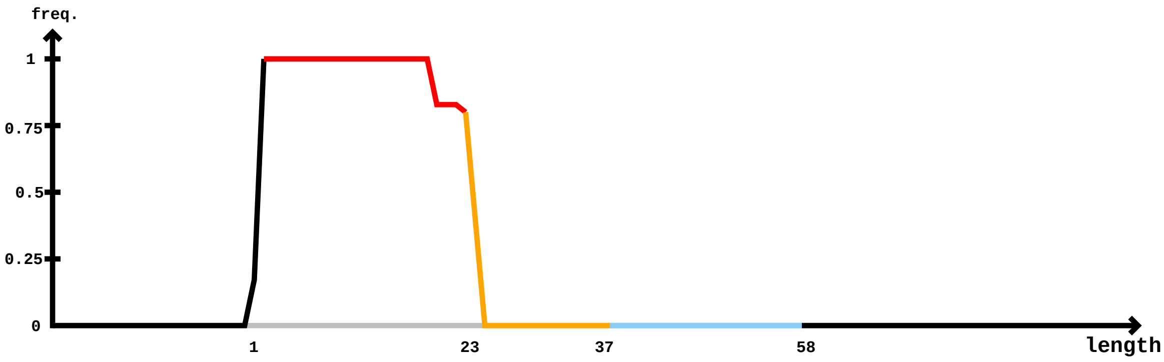


```
                          Mature                                          Star
5'- uaauauauuggguuggcaaaaaaaguccguuuggguuuuucaguaagauauuacagaaaaauccaaccaauuucuuggccaacccaguacuaugauauauuuaauagcuuu   -3'    exp
    .....((((((((((((..((.((((..(((.(((((((((.((((....)))).))))))))))))..)))).)).))))))))))))((((..........))))....    reads     mm        sample
    ...................aaaaaguUcguuuggguuu.........................................................................    6         1         seq
    ....................aaaaguccguuuggguuuuuc......................................................................    1         0         seq
    ....................aaaaguUcguuuggguuuuuca.....................................................................    28        1         seq
```
